# Microencapsulated macrophages releases conditioned medium able to prevent epithelial to mesenchymal transition

**DOI:** 10.1080/10717544.2017.1413449

**Published:** 2017-12-17

**Authors:** Anna Sola, Laura Saenz del Burgo, Jesús Ciriza, Rosa Maria Hernandez, Gorka Orive, Jorge Martin Cordero, Priscila Calle, Jose Luis Pedraz, Georgina Hotter

**Affiliations:** ^a^ Biomedical Research Networking Center in Bioengineering, Biomaterials and Nanomedicine (CIBER-BBN) Barcelona Spain; ^b^ NanoBioCel Group, Laboratory of Pharmaceutics, School of Pharmacy, University of the Basque Country (UPV/EHU) Vitoria-Gasteiz Spain; ^c^ Department of Experimental Pathology, Instituto de Investigaciones Biomédicas de Barcelona, Spanish Research Council (IIBB-CSIC, IDIBAPS) Barcelona Spain

**Keywords:** Cell microencapsulation, biomaterials, alginate, drug delivery, renal failure, mesenchymal stem cells

## Abstract

Epithelial to mesenchymal transition (EMT) has emerged as a key process in the development of renal fibrosis. In fact, EMT-derived fibroblasts contribute to the progression of chronic renal disease. In addition, anti-inflammatory M2 macrophages have exhibited a great influence on renal fibrosis. However, because of the high impact that the inputs of different environmental cytokines have on their phenotype, macrophages can easily lose this property. We aim to known if microencapsulated macrophages on M2-inducing alginate matrices could preserve macrophage phenotype and thus release factors able to act on epithelial cells to prevent the epithelial differentiation towards mesenchymal cells. We reproduced an *in vitro* model of EMT by treating adipose-derived stem cells with all-trans retinoic acid (ATRA) and induced their transformation toward epithelia. Dedifferentiation of epithelial cells into a mesenchymal phenotype occurred when ATRA was retired, thus simulating EMT. Results indicate that induction of M2 phenotype by IL-10 addition in the alginate matrix produces anti-inflammatory cytokines and increases the metabolic activity and the viability of the encapsulated macrophages. The released conditioned medium modulates EMT and maintains healthy epithelial phenotype. This could be used for *in vivo* cell transplantation, or alternatively as an external releaser able to prevent epithelial to mesenchymal transformation for future anti-fibrotic therapies.

## 1. Introduction

Traditionally, macrophage infiltration has been correlated with the severity of kidney damage and loss of function (Cao et al., 2015). Nevertheless, macrophages that uptake apoptotic cells have anti-inflammatory properties that may contribute to resolution of inflammation and tissue repair (Cao et al., 2015). The different functions of macrophages are due to changes in their phenotype. Broadly speaking, *in vitro* studies have classified macrophages as classically activated macrophages (M1 macrophages) and alternatively activated macrophages (M2 macrophages), based on their mechanism of activation and function (Wang et al., 2014). M1 phenotype is involved in the initial phase of inflammation and has tissue-damage pro-inflammatory functions. In contrast, M2 phenotype is activated in response to Th2 cytokines and mostly produces anti-inflammatory cytokines and wound healing.

M1 and M2 macrophages are main characters in the fibrotic process, chiefly preventing epithelial to mesenchymal transition (EMT) (Kushiyama et al., 2011; Pan et al., 2015). EMT, a process that make fully differentiated epithelial cells undergo transition to a fibroblast phenotype, has emerged as one important pathway in the development of renal fibrosis (Kalluri & Neilson, 2003; Liu, 2004; Grande & Lopez-Novoa, 2009). EMT is an orchestrated, highly regulated process that includes four key steps: loss of epithelial cell adhesion, *de novo* alpha1 smooth muscle actin expression and actin reorganization, disruption of tubular basement membrane, and enhanced epithelial cell migration and invasion (Liu, 2004). Cells in EMT acquire mesenchymal migratory capacity and travel across the disrupted tubular basement membrane into the interstitial microenvironment (Zeisberg & Kalluri, 2004). EMT-derived fibroblasts within the interstitium contribute to the progression of chronic kidney disease by facilitating deposition of interstitial extracellular matrix.

The use of transplanted macrophages, pumping out active factors directly at the site, has proven to be an emergent technology (Jung et al., 2012) in order to restore kidney function or modulate fibrosis through its role on EMT. However, macrophages, when transplanted *in vivo*, can lose their beneficial properties, depending on the environments (Stout et al., 2005). Thus, to preserve the macrophage regenerative effects, we propose to isolate cells by surrounding them with a semipermeable polymeric membrane. The encapsulation of macrophages will allow the nutrients to go inside the capsule and the exit of therapeutic products, thereby allowing a sustained delivery of the molecule(s) secreted by macrophages. Moreover, this encapsulation approach will isolate the enclosed macrophages from the host immune system, preventing the recognition of the immobilized cells as foreign (Ciriza et al., 2015; Orive et al., 2010). In this sense, the microencapsulation of anti-inflammatory macrophages could be a feasible option in order to avoid the change on the phenotype of these cells.

We aim to known if microencapsulated macrophages on M2-inducing alginate matrices could preserve macrophage function and thus release factors able to act on epithelial cells to prevent the epithelial differentiation towards mesenchymal cells. For this purpose, we will first test if macrophage encapsulation is advantageous in inducing and maintaining M2 phenotype and if the supernatant of encapsulated macrophages might play a role in the determination of the epithelial lineage commitment of adipose-derived stem cells (ASC) in a model previously published by our group. For this purpose, we will test if induction of M2 phenotype by IL-10 in the capsule produces higher amount of anti-inflammatory cytokines than those produced by non-encapsulated macrophages, and if this supernatant is able to modulate EMT.

Our results show that encapsulated M2 macrophages play a crucial role in the maintenance of polarized epithelia and in the prevention of EMT.

## 2. Methods

### 2.1. Cells and culture conditions

Raw 264.7 cells obtained from ATCC (Catalog number TIB-71TM, Manassas, VA) were grown in T-flasks with Dulbecco’s modified Eagles’s medium (DMEM-F12, Gibco, Waltham, MA) supplemented with 10% heat-inactivated fetal bovine serum (FBS) (Gibco) and 1% penicillin/streptomycin solution (Gibco) under standard conditions, maintained at 37 °C in a humidified 5% CO2/95% air atmosphere and passed every 2–3 days (highest passage 20).

### 2.2. Cell microencapsulation

Raw 264.7 cells were encapsulated into non-homogeneous alginate-poly-L-lysine-alginate (APA) microcapsules prepared using an electrostatic droplet generator (Nisco Engineering AG, Zurich, Switzerland). Briefly, cells were harvested from monolayer cultures using a cell scraper and suspended at a density of 4 × 10^6^ cells/ml in 1.5% (w/v) low-viscosity high gluluronic acid alginate (LVG) (NovaMatrix/FMC Biopolymer Corporation, Oslo, Norway) dissolved in a 1% mannitol solution and previously filtered through a 0.20 µm syringe filter (Millipore, Madrid, Spain). This suspension was extruded through a sterile 0.17 mm inner diameter needle using a 10 ml sterile syringe with a peristaltic pump into a calcium chloride solution (55 mM). The resulting alginate beads were maintained in agitation for 10 min in this solution. Then, the microcapsules were successively chemically cross-linked with poly-L-lysine 0.05% (w/v) for 5 min (PLL; MW 15.000–30.000 Da; Sigma-Aldrich, St. Louis, MO) and then coated with LVG-alginate 0.1% (w/v) for 5 min. Microcapsules were prepared at room temperature and under sterile conditions. Subsequently, they were maintained under normal Raw 264.7 culture conditions.

### 2.3. Characterization of microcapsules

All produced APA microcapsules were characterized in terms of morphology by microscopic observation using an inverted optical microscope (Nikon TMS, Tokyo, Japan) equipped with a camera (Sony CCD-Iris, Tokyo, Japan).

### 2.4. Cell viability assay

Cell viability of microencapsulated Raw 264.7 cells in APA microcapsules was analyzed with the LIVE/DEAD^®^ Viability/Cytotoxicity Kit (Invitrogen by Life Technologies, Carlsbad, CA). Briefly, capsules were washed for four times in test tubes with 1000 volumes of DPBS with Ca +2 and Mg +2. Then, they were mixed with the optimal studied concentration of dies (0.5 µM calcein AM and 0.5 µM ethidium homodimer-1 in DPBS), placed on 96-well plates and incubated at room temperature for 45 min in the dark. All samples were observed under a Nikon TMS microscope (calcein excitation/emission 495/515 nm; ethidium excitation/emission 495/635 nm). At least three independent experiments were analyzed for each condition.

### 2.5. Metabolic activity assay

For the determination of the metabolic activity of encapsulated Raw 264.7 cells, 10 µl of the Cell Counting Kit-8 CCK-8 solution (Sigma-Aldrich, St. Louis, MO) was added to a known number of microcapsules placed in a 96-well cell culture cluster with 100 µl of media. At least six wells were placed for each condition. Plates were incubated inside a humidified chamber for 4 h at 37 °C and read on an Infinite M200 TECAN microplate reader at 450 nm with 690 nm as the reference wavelength. At least three independent experiments were analyzed for each condition.

### 2.6. IL-10 treated cells with or without encapsulation

In order to test if encapsulation could modify the ability of IL-10 to induce the anti-inflammatory state, we studied the profile of cytokine release during 14 days in the following conditions:

- 2 ng/ml IL-10 addition to the media culture, before microencapsulation.
- 2 ng/ml IL-10 in the alginate-cell suspension just the moment before encapsulation so it remained in the capsule matrix.
- 2 ng/ml IL-10 addition to the media culture without encapsulation.

Conditioned media from encapsulated and non-encapsulated cells were analyzed for IL-10 and TNF-alpha (both from Peprotech) production by ELISA. Briefly, 100 µl of capsules was cultured in 1 ml of media, and conditioned media were collected after 24 h every 2–3 days throughout the period of study.

### 2.7. Isolation and culture of mesenchymal stem cells from mouse adipose tissue

A male Swiss albino mouse (strain CD1, Charles River Laboratories) of 3 months old and 40 g weight was used for the excision of subcutaneous adipose tissue and subsequent isolation of mesenchymal stem cells (ASC). For the extraction of adipose tissue, an incision was made in the abdominal skin and the skin was pulled away leaving the subcutaneous adipose tissue on the muscle layer. Briefly, inguinal subcutaneous fat pads were dissected and washed with 3 ml of phosphate buffered saline (PBS) containing 200 U/mL of penicillin, 200 µg/mL streptomycin (2% P/S) and 0.5 µg/ml amphotericin B (Gibco, Life Technologies, Carlsbad, CA). After debris removal, the adipose tissue was minced with a scalpel in 1 ml of digestion buffer. The digestion buffer consisted of 0.25 g bovine serum albumin (BSA) (Sigma-Aldrich, Life science, St. Louis, MO) and 0.0066 g calcium chloride (Sigma-Aldrich) dissolved in 15 ml of sterile PBS. Consequently, the minced adipose tissue was digested with 5 mg/ml collagenase type IA (Sigma-Aldrich) in a final volume of 3 ml digestion buffer, for one hour at 37 °C and 160 rpm. Collagenase digestion was stopped with 3 ml of culture medium consisted of Dulbecco's Modified Eagle's medium with 1000 mg/l glucose (DMEM-low glucose) (Sigma-Aldrich) supplemented with 10% fetal bovine serum (FBS) and 100 U/ml of penicillin, 100 µg/ml streptomycin. An additional 17 ml of culture medium was added to centrifuge at 400*g* for 10 min and the supernatant was aspirated to discard any floating adipocytes. The resultant pellet, containing the stromal vascular fraction (SVF), was resuspended in 15 ml of culture medium. To remove endothelial cell clumps the resuspended SVF pellet was passed through a 70 μm cell strainer (BD Bioscience, San Jose, CA) and collected into a 50 ml conical tube. The filtered cells were then centrifuged a second time at 400*g* for 7 min, to recover SVF cells, the resulting pellet was resuspended in 2 ml of culture medium, cultured in a T75 plastic flask (Greiner Bio-one, Kremsmünster, Austria) and incubated at 37 °C, 5% CO2. After 48 h, tissue debris and non-adherent cells were eliminated by replacing the culture medium. Subsequently, culture medium was changed every 2–3 days to prevent plastic adherence of non-ASCs and hematopoietic cells. The remaining plastic-adherent cells were further expanded and passaged before reaching 80% confluence by enzymatic dissociation using 0.25% trypsin-EDTA (Gibco, Life Technologies). The ASC isolated by culture expansion of plastic adherent cells until passage 3 were used to initiate the experiment.

### 2.8. Mesenchymal to epithelial differentiation

For *in vitro* differentiation of mesenchymal stem cells into epithelia-like cells, ASC at passage 3 were seeded into six-well plates (26,000 cells/well) in a volume of 2 ml culture medium (DMEM-low glucose +10% FBS +1% P/S) and incubated overnight at 37 °C, CO2 5% to allow plastic-adherence. Afterwards, the cells were cultured in the medium supplemented with all-trans retinoic acid (ATRA) (Sigma-Aldrich) at a final concentration of 5 µM for a total duration of 7 days. ATRA concentration and duration were determined optimal base in previous studies (Ventayol et al., 2014) as well as earlier trials. Culture medium replacement was every three days and daily morphologic changes were assessed by light microscopy to determine the frequency of ATRA administration.

### 2.9. Epithelial dedifferentiation (ATRA withdrawn)

ASC were treated with ATRA for 7 days; at day 7, the medium supplemented with ATRA was withdrawn and replaced by standard medium for 24 h. ATRA stock was prepared by dissolving the compound in 95% ethanol to a final concentration of 10 mM and was storage at −80° C protected from light.

### 2.10. Effect of macrophages conditioned media on preventing epithelial-like cell dedifferentiation

ASC were treated with ATRA for 7 days; at day 7, the medium supplemented with ATRA was withdrawn and replaced by M2 conditioned medium for 24 h.

### 2.11. RNA isolation of ASCs

Cells were harvested by mechanical dissociation and centrifuged at 1000 rpm for 5 min in an RNase free eppendorf, in order to obtain the cells pellet for RNA isolation. Total RNA was isolated using Trizol reagent (Invitrogen) according to the manufacturer's protocol. First, each sample pellet was lysed by 500 µl of trizol reagent and homogenized. Following homogenization, three phases were separated (RNA, DNA, and proteins) by adding 100 µl of chloroform and centrifuged at 12,000*g* for 15 min at 4 °C. RNA was localized only in the aqueous phase and transfer to a new RNase-free eppendorf. Then, the RNA was precipitated by adding 250 µl of isopropylalcohol and centrifuged at 12,000*g* for 10 min. After the centrifugation, the supernatant was removed and RNA was washed once with 500 µl 75% ethanol at 7500*g* for 5 min at 4 °C. The resulting pellet was dried to eliminate the ethanol, and then resuspended in 30 µl of RNase-free water.

### 2.12. cDNA synthesis and real-time reverse transcription PCR (RT-qPCR)

Complementary DNA (cDNA) was synthesized using the iScript cDNA Synthesis Kit (Bio-Rad, Hercules, CA) following the manufacture protocol. A total of 1 µg of RNA was reverse transcribed to a final volume of 20 µl cDNA, the conditions were optimized using a MyCycler (Bio-Rad) thermal cycler. The analysis of RT-qPCR were performed on an iCycler iQ RT-qPCR detection system (Bio-Rad) using either SYBR Green Supermix (Bio-Rad) or pre-validated Taqman probes, depending on the primer used following the manufacture protocol (see Table 1). The samples were analyzed each time by triplicate and the results were normalized to the housekeeping gene glyceraldehyde 3-phosphate dehydrogenase (GAPDH).

**Table 1.** Primer sequence.

Gen	Source	Primer sequence
PAX2 (NM.011037.4)	Applied biosystems	Taqman Gene Expression Assay ID: Mm01217939_m1
Aquaporina 1 (NM 007472.2)	Invitrogen	Reverse: GGGACTTCCTCTCCCTCAAA Forward: AAGTCCCCCTCACTCCAAAG
Megalina (NM 001081088.1)	Invitrogen	Reverse: TGCTGGCTTGGAAGACTTTT Forward: GGTTCGTTATGGCAGTCGTT

### 2.13. Immunostaining techniques

#### 2.13.1. Immunofluorescence of actin fibers stained with phalloidin

Cells were grown on 15 mm glass coverslips, as previously described in the mesenchymal to epithelial differentiation protocol. At the end of the experiments, the samples cells were washed twice with PBS (5 min each) and fixed with 4% formaldehyde solution in PBS for 10 min at room temperature (RT). Fixed cells were washed with PBS, permeabilized with acetone for 5 min at 4 °C, and washed again with PBS. Thereafter, the cells were blocked with normal goat serum (NGS) (Gibco, Life Technologies) for 60 min to reduce nonspecific binding of the antibody. Briefly, the preparations were stained with Alexa Fluor 568 phalloidin (Molecular Probes, Eugene, OR) at 1:40 dilution in 1% BSA/PBS for 30 min at RT protected from light; then, washed with PBS and counterstained with 0.2 mg/ml DAPI solution (Sigma-Aldrich) for 5 min, to visualize the nuclei.

#### 2.13.2. Immunofluorescence of vimentin

The above protocol was used to fix, permeabilize, and block. Then, the cells preparations were incubated with anti-vimentin antibody, mouse monoclonal (clone V9, Sigma) at 1:1000 dilution in PBS with 10% NGS and 2% FBS, for one hour at RT. Followed by the incubation of goat anti-mouse IgG secondary antibody (Sigma) at a dilution 1:2000 for one hour at RT protected from light. Nuclei were stained with DAPI.

#### 2.13.3. Immunofluorescence of megalin

The same initial protocol was applied to fix and permeabilize, but this time FBS (Gibco, Life Technologies) was used to block in order to avoid unspecific binding because the primary antibody was produced in goat specie. After washing, the preparations were incubated with megalin antibody (P-20), goat polyclonal (Santa Cruz Biotechnology, Dallas, TX) at 1:500 dilution in 2% FBS/PBS for one hour at RT followed by the incubation of anti-goat IgG (H + L) secondary antibody, Alexa Fluor 488 conjugate (Molecular Probes) at 1:1000 dilution in PBS for 30 min at RT protected from light. Nuclei were stained with DAPI.

#### 2.13.4. Immunocytochemistry cytokeratin 18 (CK18)

The cells seeded in coverslips were fixed in 4% formaldehyde/PBS for 15 min at RT, washed twice with PBS and permeabilized with acetone for 5 min at 4 °C. They were washed again and blocked with 1% BSA in PBST (PBS +0.1% Tween 20) for 30 min at RT. After two more washes with PBS, the preparations were incubated with anti-Cytokeratin 18 antibody, rabbit monoclonal (clone E431-1, Millipore) in a dilution 1:50 in 1% BSA/PBST overnight followed by the incubation of biotinylated goat anti-rabbit IgG (H + L) secondary antibody (Vector Laboratories, Burlingame, CA) at a 1:100 dilution for one hour. After four washes with PBS, the cells were incubated with peroxidase-conjugated avidin (Invitrogen) for one hour in humid chamber and stained with three, 3′-diaminobezidine (DAB) peroxidase substrate kit (Vector Laboratories) for 10 min at RT and, after washing, counterstained with hematoxylin.

### 2.14. Statistical analysis

Data are presented as mean ± S.D. Statistical analysis was performed by unpaired Student’s *t*-test between two groups using GraphPad Prism version 6.0c. software (La Jolla, CA). *p* < .05 was considered as statistically significant.

### 2.15. Figure preparation

The images were prepared for publication using Adobe Photoshop software (Adobe Systems, San Jose, CA) without modifications.

## 3. Results

### 3.1. Microencapsulated macrophages are able to survive and proliferate during the length of the study

**Figure 1.**
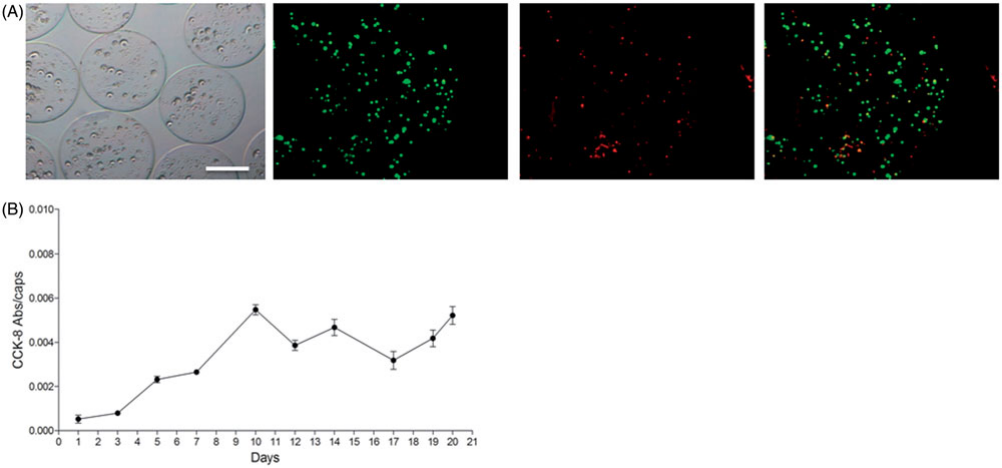
(A) Morphology of microencapsulated Raw 264.7 cells at 4×10^6^ cell/ml density under the inverted optical microscopy (left panel) and fluorescence microscopy after calcein/ethidium staining the day after encapsulation. The last image on the right corresponds to the merge image of both staining. Scale bar 200 μm. (B) Metabolic activity of encapsulated Raw 264.7 cells measured by the Cell Counting Kit 8 (CCK-8) assay for three weeks. *N* = 3. Data are means ± SD..

Raw 264.7 macrophages were encapsulated in alginate-poly-L-lysine microcapsules (APA) at a 4 × 10^6^ cell/ml density and first, the physical appearance and morphology of the scaffolds were assessed under the microscopy. Microcapsules were completely spherical, with a mean diameter of 370 ± 10 µm and had a homogeneous surface (Figure 1(A)). These characteristics are very important because the *in vivo* biocompatibility of these devices relies on factors such as the quality of the material used for the matrix as well as their smoothness (de Vos et al., 1994; Ponce et al., 2006).

Then, the viability of the microencapsulated macrophages was analyzed over the course of the study (21 days) with the viability/cytotoxicity assay or calcein/ethidium staining, which was performed twice a week. Importantly, no signs of cellular lysis or necrosis were observed during this time lapse. As seen in Figure 1(A), most cells appeared stained in green (alive) even the day after microencapsulation, when usually some cells might die because of the procedure. In addition, the metabolic activity of the enclosed cells was analyzed during those three weeks as another indication of their physiological state (Figure 1(B)). During the monitorization, it could be observed that cells progressively increased their metabolic activity during the first 10 days and, afterwards, the activity remained more stable until the end of the study. Therefore, Raw 264.7 cells seemed to adapt correctly to the new microenvironment provided by the alginate matrix. In addition, the external membrane of the APA microcapsules seemed to control correctly the oxygen and nutrient availability. Thus, macrophage Raw 264.7 cells entrapped in alginate scaffolds were able to survive and proliferate.

### 3.2. Encapsulation could modify the ability of IL-10 to induce the anti-inflammatory state, enhance the metabolic activity and the viability of the culture

**Figure 2.**
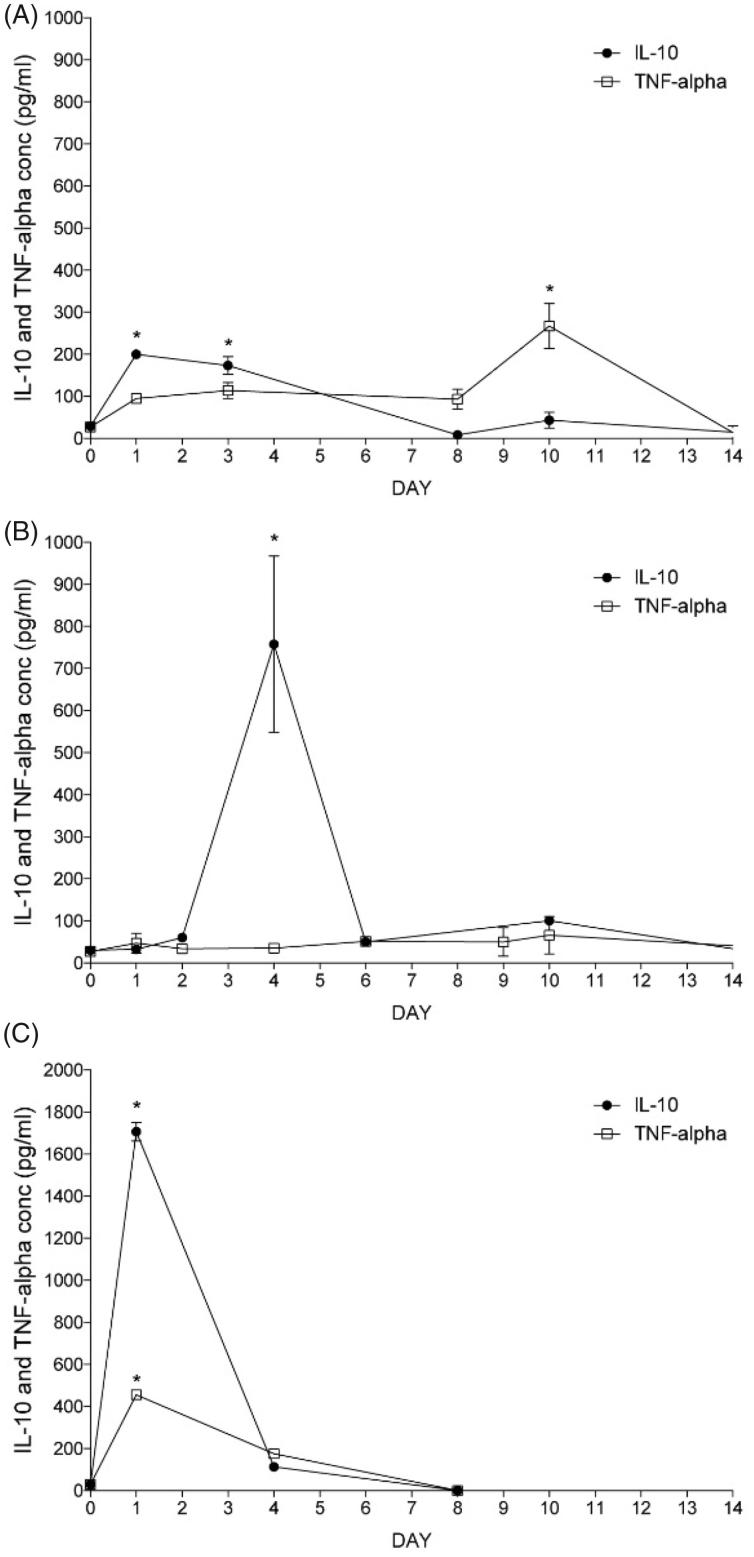
Induction of the anti-inflammatory M2 state of Raw 264.7 cells with or without microencapsulation. IL-10 and TNF-alpha production of Raw 264.7 cells determined by ELISA when 2 ng/ml of IL-10 is added into the culture media before encapsulation (A), in the alginate matrix (B), or without encapsulation but with IL-10 addition once (C). *N* = 3. Data are means ± SD, *p < .05 versus control basal levels.

IL-10 is one of the cytokines that can induce the M2 state on macrophages (Jung et al., 2012). Therefore, we added it to media culture where cells were cultured before microencapsulation. In addition, we included this cytokine in the alginate-cell suspension just the moment before encapsulation so it is kept in the capsule matrix (Figure 2). In addition, we studied the effect of IL-10 addition in cell culture without encapsulation.

In order to know which of these three conditions induced on Raw 264.7 cells an anti-inflammatory state, we studied the profile of cytokine release by each group.

When cells were treated previously to encapsulation, IL-10 levels on the conditioned media increased significantly respect to basal value during the first days after encapsulation (Figure 2(A)). However, this production sharply decreased after the first week giving way to a significant increase on the TNF-alpha production during the second week (Figure 2(A)). Afterwards, both levels decreased significantly.

When cells were encapsulated in IL-10-enriched alginate microcapsules, we observed higher increase in IL-10 from day 1, reaching the highest significant levels on day 4 (Figure 2(B)). Afterwards, they both decreased almost at the same pace. In the meanwhile, TNF-alpha (Figure 2(B)) remained stable with no significant differences between them and the control values during the length of the study. Thus, indicating that IL-10 enriched alginate capsules induces release on anti-inflammatory cytokines and does not alter the inflammatory TNF.

When cells were not encapsulated but stimulated once with IL-10, IL-10 levels increase significantly with respect to basal values, reflecting initial IL-10 addition, and decrease sharply until the end of the study (Figure 2(C)). TNF-alpha values also increased significantly (Figure 2(C)).

**Figure 3.**
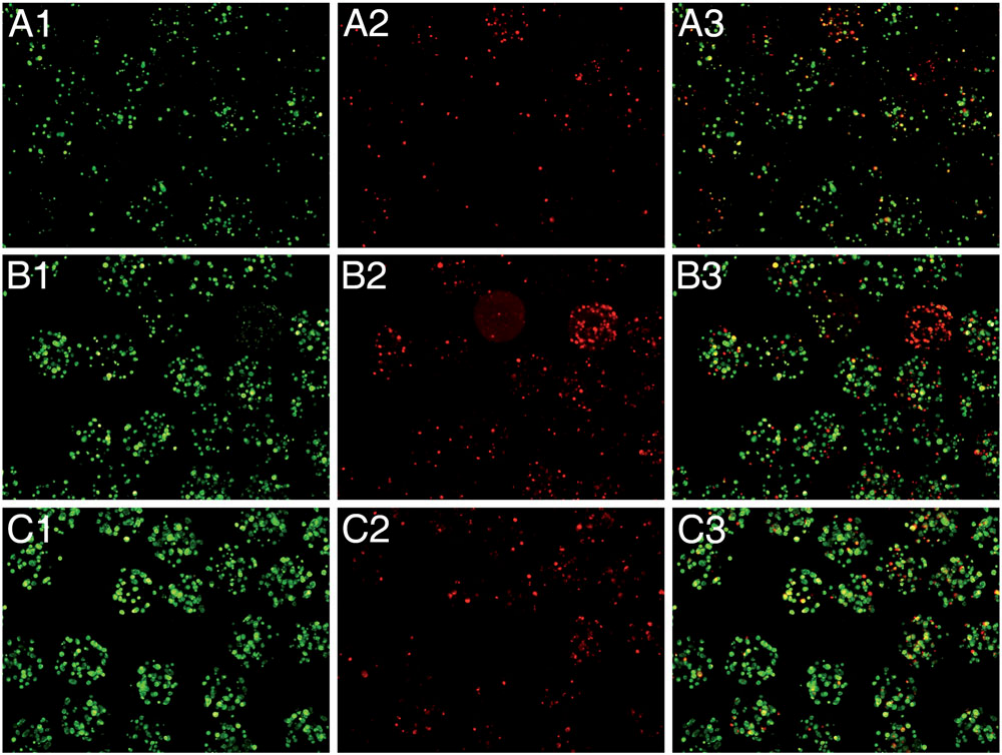
Fluorescence microscope images of encapsulated Raw 264.7 cells stained with calcein AM (live cells, column 1) and ethidium homodimer (dead cells, column 2) at day 4 post-encapsulation. Control capsules (A), 2 ng/ml of IL-10 added into the culture media before encapsulation (B), or 2 ng/ml of Il-10 added in the alginate matrix (C). Column 3 represents the merged image from columns 1 and 2.

As a qualitative measurement of the viability of encapsulated Raw 264.7 macrophages on the different conditions described, we performed the calcein/ethidium staining twice a week (Figure 3). As it can be observed, when IL-10 is added either into the alginate matrix or in the culture media before encapsulation, cells are considerably more alive (more cells appear in green) (see C rows (IL-10 in the matrix) and B rows (IL-10 before encapsulation) versus A (controls)).

In the figure, it can be observed how the viability of encapsulated cells slightly increased when IL-10 was added before or in the alginate matrix, with respect to encapsulated non-treated cells. Therefore, under our point of view, the most suitable approach for inducing an anti-inflammatory state on the cells is the addition of IL-10 directly to the capsule matrix. As seen on the C row, more cells were alive and at the same time, there is not an increment on the number of ethidium stained cells.

Thus, the encapsulation of macrophages in an IL-10 enriched matrix is the best condition of encapsulation in terms of anti-inflammatory (high IL 10 and low TNF-alpha release) profile, in terms of viability.

### 3.3. Microencapsulated macrophages conditioned media prevents epithelial-like cell dedifferentiation

**Figure 4.**
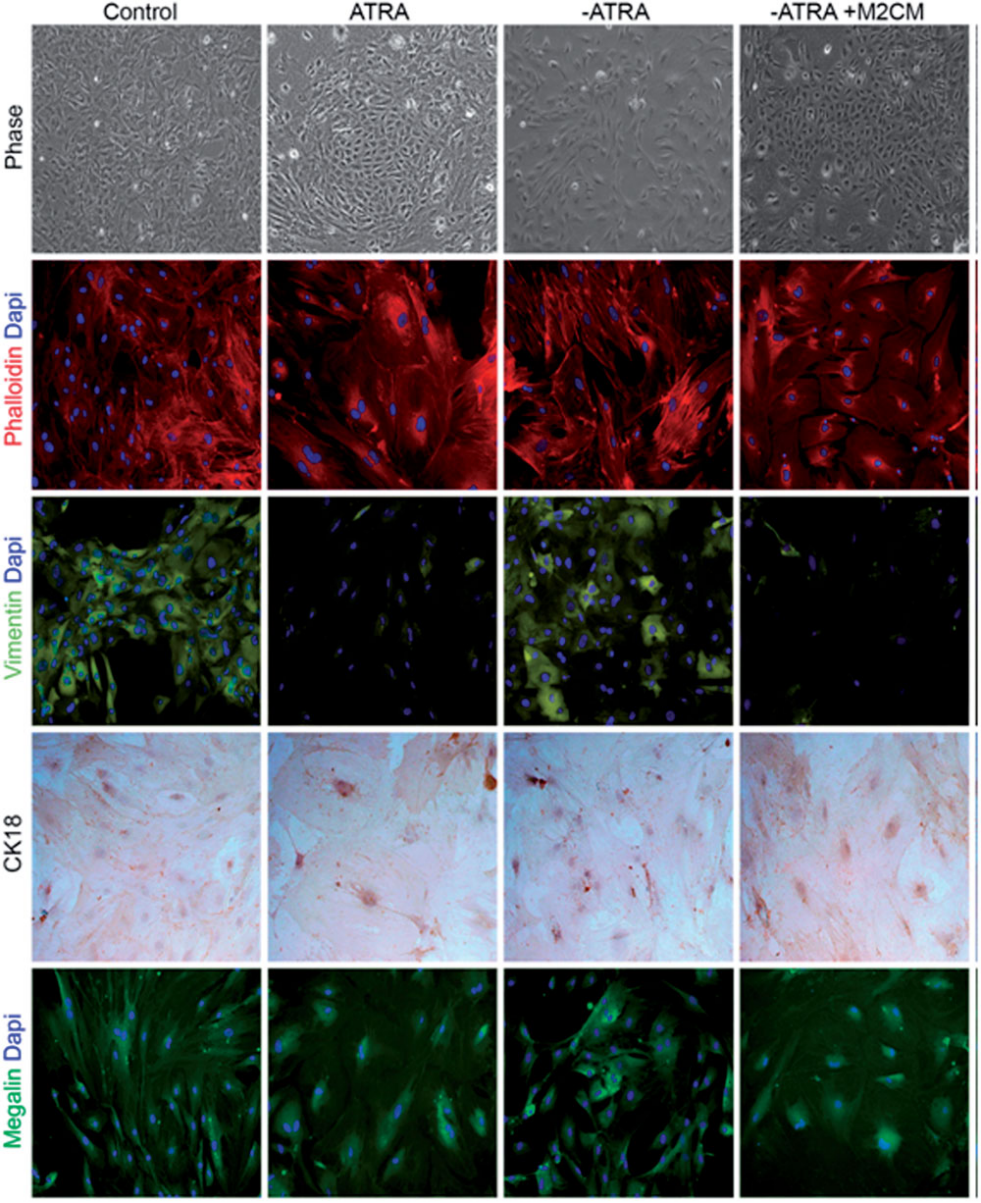
Effect of anti-inflammatory M2 supernatants on epithelial to mesenchymal transition markers. Phase contrast images and immunofluorescence of phalloidin, vimentin, megalin, and cytokeratin18 (CK-18) in ASC untreated (Control); ASC treated with ATRA for 7 days (ATRA); below ATRA withdrawal for 24 h (−ATRA); ATRA withdrawal followed by anti-inflammatory macrophages conditioned medium for 24 h following (−ATRA + M2CM)), Phase-contrast images and phalloidin staining of F-actin cytoskeleton (red) nuclei (blue). In addition, immunofluorescence of vimentin (green), nuclei (blue). Immunocytochemistry of CK18, nuclei counterstained with hematoxylin. Immunofluorescence of megalin (green), nuclei (blue). Images were obtained by phase contrast microscopy, immunofluorescence, and immunocytochemistry. Magnification 20×.

Having established the best anti-inflammatory conditions of microencapsulated macrophages, we tested the ability of conditioned media in the determination of the epithelial lineage commitment of ASC in a model previously published by our group. Phase-contrast microscopy analysis of ASC cultured with 5 mM ATRA for 8 days showed a conversion into epithelial polygonal phenotype with respect to untreated controls (Figure 4, upper panel). A flattened fibroblast-like morphology was observed in untreated cells, whereas ATRA stimulation gradually changed cell morphology into an epithelial, cobblestone-like phenotype, as described previously by our group (Ventayol et al., 2014). Changes in the organization of actin fibers by immunofluorescence staining using phalloidin also revealed that ATRA stimulation presented a more organized distribution with a staining pattern on nucleus and membrane (Figure 4, second line). In addition, imnunofluorescence of the mesenchymal marker vimentin was decreased (Figure 4, third line), while the immunofluorescence of the epithelial marker CK18 (Figure 4, fourth line), represented by an increase in brown color, showed that ATRA treated cells were CK18 positive.

Induced epithelial-like cells were deprived of their differentiation inductor ATRA for 24 h. The morphology and distribution were similar to a mesenchymal phenotype, indicating that epithelial-like cells de-differentiate into their mesenchymal origins upon ATRA withdrawal. These cells showed themselves as vimentin+/CK18− with an expression of megalin that also correlated to a mesenchymal phenotype.

At this point, we stimulated the cells with conditioned media (M2MC) (medium obtained at day 4, showing an anti-inflammatory phenotype corresponding to macrophages encapsulated in IL-10-enriched matrixes) and, interestingly, conditioned medium was able to differentiate ASC into epithelial cobblestone-like cells, resembling those cells treated with ATRA for 8 days. Consistent with this, the expression patterns of epithelial and mesenchymal markers were identified as vimentin−/CK18+; megalin expression also determined an epithelial phenotype.

**Figure 5.**
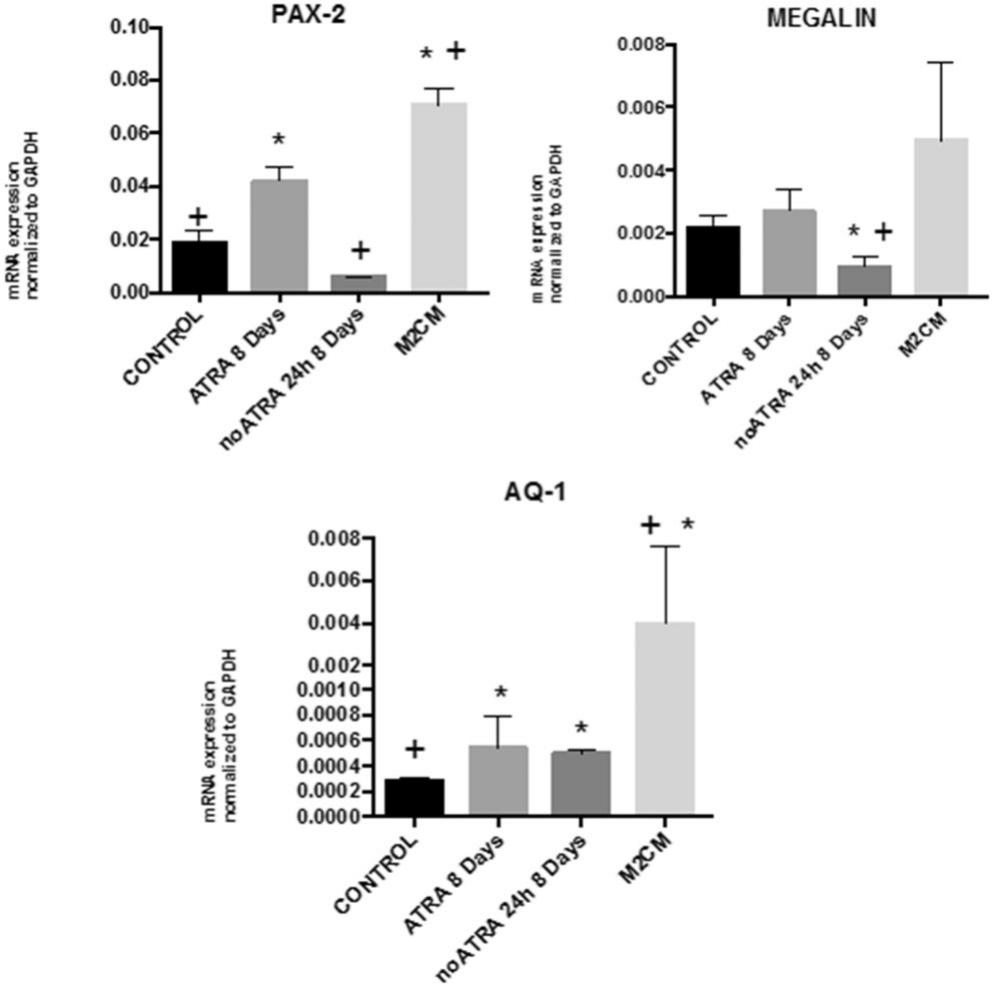
qRT-PCR analysis of PAX-2, MEGALIN, and AQUAPORIN-1. Total RNA was extracted from ASC untreated (Control); ASC treated with ATRA for 7 days (ATRA), below ATRA withdrawal for 24 h (−ATRA), treated with anti-inflammatory macrophages conditioned medium for 24 h following ATRA withdrawal (−ATRA + M2CM). *N* = 4. Data are means ± SD. *p < .05 versus untreated. +*p* < .05 versus ATRA.

To further confirm these results, the mRNA expression of the epithelial markers PAX 2, megalin, and aquaporin was quantified by RT-qPCR. Overexpression of the markers was observed in ASCs cultured with ATRA, thus indicating that ATRA induces the differentiation of ASCs into epithelial-like cells (Figure 5).

Down regulation of the marker, expression was observed with ATRA withdrawal, thus indicating that ATRA withdrawal induces de-differentiation towards a mesenchymal phenotype (Figure 5). The mRNA expression of the epithelial markers PAX 2, megalin, and aquaporin-1 was significantly increased in cells treated with M2CM, thus indicating the effect of M2 conditioned media (M2CM) on promoting epithelial phenotype (Figure 5).

Thus, results indicate that M2CM is able to preserve epithelial differentiation to mesenchymal phenotype and prevent EMT.

## 4. Discussion

Anti-inflammatory M2 macrophages have shown to reduce inflammation-associated renal damage (Wang et al., 2007). However, because of their functional adaptability, when transplanted *in vivo*, they could lose their anti-inflammatory and regenerative profile. In this sense, although the cell microencapsulation technology has been used mainly as a way of protecting therapeutic factors producing cells from the host immune system when implanted *in vivo* (Acarregui et al., 2014; Gurruchaga et al., 2015; Saenz del Burgo et al., 2017), alginate microcapsules have also been used for the *ex vivo* production of different substances such as bispecific antibodies for cancer immunotherapy (Saenz del Burgo et al., 2015). Therefore, this second strategy was tested for the induction and maintenance of macrophages on a M2 anti-inflammatory state in order to obtain a cocktail of therapeutic substances that were able to keep the morphology of healthy epithelial cells, preventing epithelial differentiation towards mesenchymal cells.

This study shows a high cell viability and metabolic activity that confirms the suitable adaptation of macrophages to the new environment provided by the polymeric microcapsule (Figure 1(A,B)). In fact, microcapsules not exceeding 400 µm in diameter are known to guarantee enough transport of oxygen and nutrients (Sugiura et al., 2005). In order to induce a M2 state, IL-10 was used in different approaches: mixing it within the alginate and cell suspension or in the culture media before encapsulation (Figure 2). Results indicate that addition of IL-10 directly in the alginate matrix is the best condition of encapsulation in terms of anti-inflammatory (high IL 10 and low TNF release) profile and in terms of viability. In fact, higher viability, anti-inflammatory cytokine IL-10, and reduced levels of the inflammatory cytokine TNF were observed with respect to the other conditions.

We decided to use this last condition as it required the addition of IL-10 just once, now the capsules are produced. On these cells, we observed high IL-10 production the first week after encapsulation, having the highest levels on day 4th and TNF remained unaltered. Thus, the conditioned media obtained on that day was used in the following experiments.

Renal epithelial differentiation of ASCs into epithelial-like cells was first observed after transplantation of human ASCs into ischemic mouse kidneys, confirmed by the expression of CK18 (Li et al., 2010). *In vitro* studies also showed that ASC differentiation toward renal epithelium could be induced by factors secreted from renal tubular epithelial cells (Baer et al., 2009, 2013). Brzoska et al. (2005) described an *in vitro* protocol using ATRA in order to differentiate ASCs into epithelial cells that expressed increased levels of the epithelial marker CK18 and decreased levels of the mesenchymal marker vimentin.

In the present study, ASCs cultured with ATRA increased the expression of the epithelial marker cytokeratin CK18, while the mesenchymal marker vimentin was reduced (Figures 4 and 5). Furthermore, the membrane glycoprotein megalin, that shows a key role in determining kidney functional responses in renal epithelial cells, was also increased. After treatment with ATRA, cells undergo significant morphological changes. The typical fibroblastic, mesenchymal-like morphology of ASCs gradually changes toward a more epithelial-like, polygonal morphology. These morphological and cytoskeletal changes represent the key process during EMT, allowing the cells to adopt the characteristic epithelial cell apical–basal polarity (Ventayol et al., 2014).

We reproduced an *in vitro* model of EMT through the administration of ATRA in ASCs. The epithelial-like cells obtained were crucial to determine the morphological and genetic changes originated during this process and assisted as a control model for monitoring the effects of ATRA withdrawal. When we deprived the epithelial-like cells from ATRA, we reversed their differentiation process; cytokeratin CK18, PAX 2, megalin, and aquaporin-1 diminished and the cells lost polarity and acquired a fibroblastic morphology (Figures 4 and 5). In this sense, we promoted the de-differentiation of epithelial cells into mesenchymal phenotype by mimicking a process of EMT.

In the past several years, EMT, a process by which fully differentiated epithelial cells undergo transition to a fibroblast phenotype, has emerged as one important pathway in the development of renal fibrosis (Kalluri & Neilson, 2003; Liu, 2004; Grande & Lopez-Novoa, 2009). EMT is an orchestrated, highly regulated process that includes four key steps: loss of epithelial cell adhesion, *de novo* alpha-smooth muscle actin expression, and actin reorganization, disruption of tubular basement membrane, and enhanced epithelial cell migration and invasion (Liu, 2004).

Evidence of the EMT process *in vivo* in the setting of kidney diseases was provided by Iwano et al. (2002) showing the most convincing evidence for EMT *in vivo* as a source of interstitial, matrix-producing fibroblasts. Using genetically tagged proximal tubular epithelial cells, they showed that up to 36% of all fibroblast-specific protein positive (FSP1-positive fibroblasts) within the interstitial space originate from renal proximal tubules after unilateral ureteral obstruction (UUO), thus indicating that a third of all disease-related fibroblasts originate from tubular epithelia at the side of injury.

In our knowledge, the developed model of EMT is novel and the role of anti-inflammatory macrophages in EMT process has never been studied earlier in this setting.

It seems that M1 and M2 macrophages are involved in the fibrotic process, but their involvement is controversial and in some studies, it depends on the fibrosis stage after injury. UUO is the most used animal model to induce fibrosis and it has been found that elimination of M2 macrophages at the advanced stage after fibrosis induction in UUO animals reduced the formation of renal fibrosis, but elimination of M1 macrophages in the early UUO stage did not modify the fibrotic features (Shen et al., 2014). In the same way, other authors indicated that M2 macrophage depletion, but not M1 macrophage depletion, specifically inhibited EMT, and subsequently inhibited renal fibrosis (Pan et al., 2015). In the other hand, others found that the increase in the proportion of M2 macrophages plays an important beneficial role in the resolution of renal fibrosis, being the most characteristic feature of the early recovery phase after UUO (Kushiyama et al., 2011). Another experimental model of UUO showed that mice expressing mannose receptor C type 2 (Mrc2), which is expressed by M2 macrophages, ameliorated renal fibrosis compared with Mrc2 deficient mice (López-Guisa et al., 2012).

Using our *in vitro* system, we confirmed that the addition of conditioned medium obtained from encapsulated anti-inflammatory macrophages (M2CM) contributes to maintain the morphology of healthy epithelial phenotype. The epithelial-like cells undergoing a mesenchymal transformation did not experience re-organization of cytoskeleton distribution and they expressed CK18 and megalin in the absence of vimentin, indicating the ability of this conditioned medium to prevent epithelial to mesenchymal transformation and to contribute to maintain healthy phenotype.

When tested the effectivity of conditioned medium obtained at day 4 (M2CM), showing an anti-inflammatory phenotype (Figure 2(C)) on ATRA depleted cells, results indicated the ability to reduce epithelium to mesenchymal transition, since increases in PAX, megalin, and aquaporin 1 were detected, but not reached statistical significance (Figure 5).

Thus, results indicate that conditioned medium from encapsulated macrophages at the maximum anti-inflammatory level preserved the epithelial transformation and contributed to maintain healthy phenotype, thus preventing EMT.

## 5. Conclusions

In conclusion, microencapsulated macrophages on M2-inducing alginate matrices releases conditioned medium that is able to prevent EMT and maintain healthy epithelial phenotype. This could be used for *in vivo* cell transplantation, or alternatively as an external system able to prevent epithelial to mesenchymal transformation for future anti-fibrotic therapies.
